# The Influence of Simulated Microgravity on Purinergic Signaling Is Different between Individual Culture and Endothelial and Smooth Muscle Cell Coculture

**DOI:** 10.1155/2014/413708

**Published:** 2014-08-28

**Authors:** Yu Zhang, Patrick Lau, Andreas Pansky, Matthias Kassack, Ruth Hemmersbach, Edda Tobiasch

**Affiliations:** ^1^Department of Natural Sciences, Bonn-Rhine-Sieg University of Applied Sciences, 53359 Rheinbach, Germany; ^2^Institute of Pharmacology and Medical Chemistry, University of Dusseldorf, 40225 Dusseldorf, Germany; ^3^Institute of Aerospace Medicine, German Aerospace Center, 51147 Cologne, Germany

## Abstract

Exposure to microgravity conditions causes cardiovascular deconditioning in astronauts during spaceflight. Until now, no specific drugs are available for countermeasure, since the underlying mechanism is largely unknown. Endothelial cells (ECs) and smooth muscle cells (SMCs) play key roles in various vascular functions, many of which are regulated by purinergic 2 (P2) receptors. However, their function in ECs and SMCs under microgravity conditions is still unclear. In this study, primary ECs and SMCs were isolated from bovine aorta and verified with specific markers. We show for the first time that the P2 receptor expression pattern is altered in ECs and SMCs after 24 h exposure to simulated microgravity using a clinostat. However, conditioned medium compensates this change in specific P2 receptors, for example, P2X7. Notably, P2 receptors such as P2X7 might be the important players during the paracrine interaction. Additionally, ECs and SMCs secreted different cytokines under simulated microgravity, leading into a pathogenic proliferation and migration. In conclusion, our data indicate P2 receptors might be important players responding to gravity changes in ECs and SMCs. Since some artificial P2 receptor ligands are applied as drugs, it is reasonable to assume that they might be promising candidates against cardiovascular deconditioning in the future.

## 1. Introduction

Exposure to microgravity conditions during space missions induces a variety of health issues in astronauts, including bone loss, muscle atrophy, decreased immune activity, and cardiovascular deconditioning [1–3]. The cardiovascular deconditioning is very likely caused by the dysfunction of the major vascular cells: endothelial cells (ECs) and smooth muscle cells (SMCs). ECs build up the monolayer coating inner surface of blood vessels. Layers of SMCs arranged in fibers support the EC monolayer by providing contraction and relaxation of vessels [4]. Importantly, the interaction between ECs and SMCs has been shown to be a key player in human cardiovascular physiology [5]. ECs are sensitive to mechanical stress, and they secret cytokines inhibiting SMC proliferation [6]. Purinergic receptors can bind extracellular nucleotides such as ATP [7, 8] and they are crucial players in regulating a series of physiological and pathological cardiovascular processes such as atherosclerosis, hypertension, and vascular pain [9, 10]. Purinergic receptors are divided into P1 receptors and P2 receptors [11]. P2 receptors can be subdivided into P2X receptors that are ion channels and P2Y receptors that are G protein-coupled receptors [12]. Until now, seven P2X (P2X1-7) and eight P2Y (P2Y1, 2, 4, 6, 11, 12, 13, and 14) have been characterized. However, the role of extracellular nucleotides on vascular cell function under microgravity condition is still unknown.

Recent publications have shown that cytoskeleton arrangement, gene expression of extracellular matrix, and cell surface adhesion molecules in ECs were altered after 22 seconds and 24 h exposure to microgravity [13–16]. ECs formed tubes after culturing for longer term (7 days) under simulated microgravity conditions using a Random Positioning Machine (RPM) [17]. On the other hand SMCs showed suppressed proliferation and an enhanced rate of apoptosis after 72 h exposure to simulated microgravity using a rotating wall vessel (RWV) [18]. However, these findings were encountered when single cell type such as endothelial or smooth muscle cells was cultured under real or simulated microgravity. Considering ECs already showed to secrete different cytokines under simulated microgravity using RPM [19], the important interactions between ECs and SMCs under microgravity condition should be evaluated and thus require investigations.

In this study, an indirect cell coculture model was established by culturing SMCs with EC-conditioned medium and vice versa. The P2 receptor expression pattern was analyzed and compared under three conditions: normal gravity control (1 g), simulated microgravity (MG), and simulated microgravity with conditioned medium. For the simulation of microgravity a fast rotating clinostat was used, in which the cells were quickly rotated around one axis perpendicular to the direction of gravity [20]. The influence of conditioned medium collected from normal gravity and simulated microgravity on cell proliferation and migration was also investigated.

## 2. Methods

### 2.1. Isolation and Characterization of Bovine Aortic Endothelial and Smooth Muscle Cells

Bovine aorta was cut longitudinal into 5 cm sections and divided again into rectangles after removing residual and connective tissues. The cut aorta into type I collagenase (10 mg/mL in PBS) coated cell culture dishes, with the inner layer (endothelium) attached to the collagenase, and incubated for 60 min at 37°C. The aortic endothelial cells were slightly scraped with a cell scraper and put onto gelatin (in PBS (1% v/v)) coated culture plates [21]. Medium was added to the freshly scraped cells and the plates were then incubated at 37°C, 5% CO_2_ under humidified conditions. Aortic smooth muscle cells were isolated by obtaining the media layer through removal of the outer layer and scraping off the endothelial cells. The media layer was cut into 2 mm × 2 mm sections and put into cell culture dish for 2 h without medium to allow these sections adhering tightly to the surface [22]. The medium was added and the pieces were incubated at 37°C, 5% CO_2_ under humidified conditions for up to a week to let SMCs migrate and proliferate from the tissue pieces to the surface of culture dish.

### 2.2. Cell Culture

The cells were cultured in DMEM medium (Merck Millipore, Berlin, Germany) supplied with 10% FCS and 1% penicillin/streptomycin. ECs and SMCs were split and seeded at a density of 5000 cells/cm^2^, after they reached a level of 80–90% confluence. ECs and SMCs with passage number 2–4 were used. The cell line human microvascular endothelial cell-1 (HMEC-1), C2C12 (ATCC number: CRL-1772) and MG-63 (ATCC number: CRL-1427), and U-87 MG (ATCC number: HTB-14) were cultured in DMEM medium and subsequently used as positive control.

### 2.3. Clinostat Experiments

**Figure 1:**
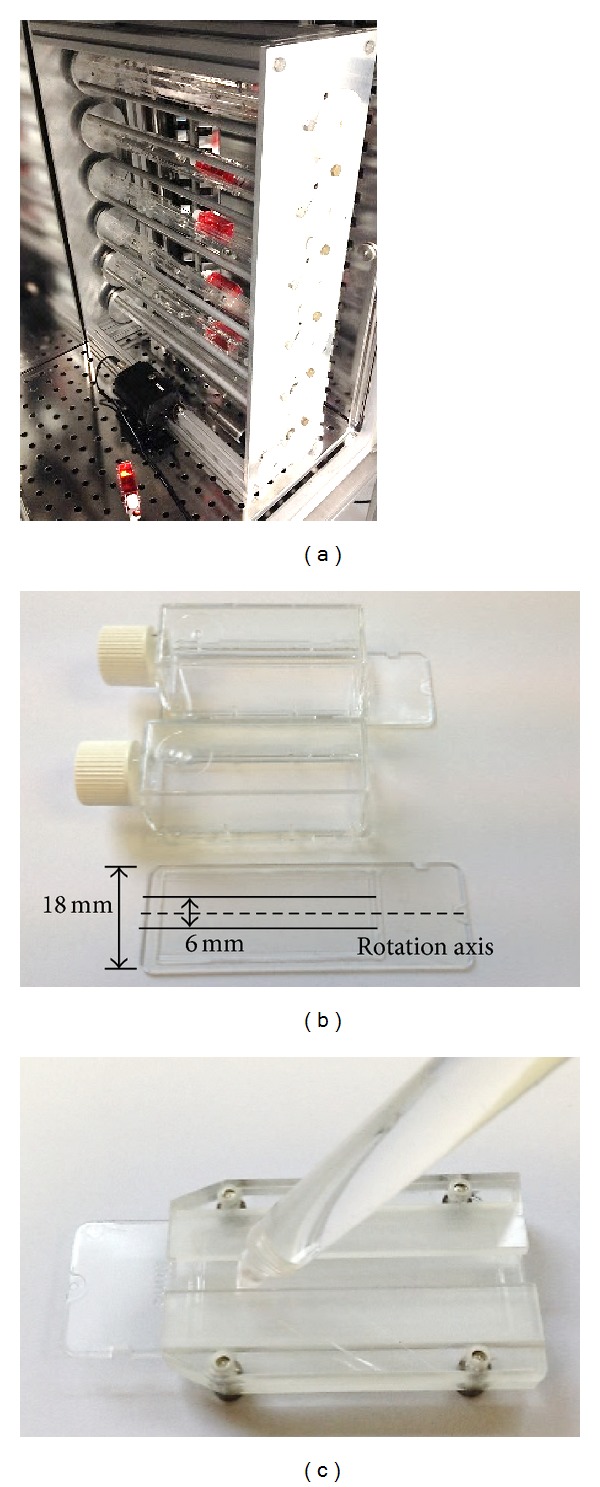
Cell Culture in a Clinostat to Simulate Microgravity Conditions. The clinostat (a) was used to simulate the microgravity environment by rotating cells. Only cells grown in the 6 mm area in the middle of culture slide (b) received the optimal simulated microgravity and were thus harvested using a special cell scraper (c).

The fast-rotating 2D clinostat used in this study was originally developed by the Institute of Aerospace Medicine, German Aerospace Center (DLR) (see Figure 1(a)). It has 6 parallel horizontal axes, each for fixation for up to 4 slide flasks. ECs and SMCs were seeded at a density of 10,000 cells/cm^2^ onto 9 cm^2^ cell culture slide flasks (Nunc, Thermo Fisher Scientific, Langenselbold, Germany). When they reached a confluence level of 60%–70%, the culture flasks were filled up completely with DMEM medium. To avoid shear stress and thus the induction of respective metabolic changes in signal transduction pathways, for example, apoptosis, air bubbles were removed carefully. The flasks were inserted on the clinostat and rotated at 60 rpm for 24 h in the CO_2_ incubator at 37°C. Controls were also filled with medium and placed simultaneously under normal gravity.

Cells from the whole flask were first used to analyze the P2 receptor expression pattern that altered subtypes could be distinguished from unaffected. Later, according to the clinostat principle, only cells exposed to minimal* g*-forces were taken for further analysis. This means that only cells from the middle of the flask were taken (see Figure 1(b)). Under a defined constant speed of 60 rpm the maximal residual acceleration at an area of 6 mm provided an optimal quality of simulated microgravity (≤0.0121 g) [23]. Thus, only cells within this 6 mm area were isolated to evaluate altered P2 subtypes for both gene and protein expression in detail. To maintain the cells accurately and consistently in the center, a special chamber consisting of two cover plates, attached to a bottom plate [23], was used to allow slide insertion without wiping off the cell layer. A corresponding cell scraper was used to scratch the cells from the specific 6 mm width area in the center (Figure 1(c)).

### 2.4. Conditioned Medium

To investigate a possible paracrine influence on P2 receptor expression, ECs and SMCs were seeded in a density of 2500 cells/cm^2^. Cell growth medium was collected when they were 80%–90% confluent. The conditioned medium (CM) was composed out of cell growth medium and normal DMEM medium in a ratio of 1 : 2 on respective cell type. The SMC-conditioned medium was subsequently fully added into the culture slide with ECs and set of a 24 h clinorotation as a group of EC MG + CM. The ECs in normal gravity group (EC 1 g) and in clinorotation but filled with normal DMEM medium (EC MG) were set simultaneously. The similar experiments were set for SMCs with normal gravity (SMC 1 g), clinorotation (SMC MG), and clinorotation filled with EC-conditioned medium (SMC MG + CM).

To evaluate a possible paracrine effect on cell proliferation and migration, cell growth medium was collected from cells cultured 24 h in normal gravity and cultured 24 h under clinorotation, respectively. The used conditioned medium (CM) was composed out of cell growth medium and normal DMEM medium in a ratio of 1 : 2 on respective cell type. The ECs were subsequently treated with normal DMEM medium, SMC-conditioned medium from normal gravity (CM SMC + 1 g), and clinorotation (CM SMC + MG) separately for proliferation or migration assays. Similar experiments were set and performed with SMCs as well. All experiments were performed with samples from three cows.

### 2.5. RNA Isolation and Semiquantitative PCR

RNA was extracted after clinorotation using a Ribozol RNA reagent (Amresco, OH, USA). cDNAs were synthesized from 2.0 *μ*g total RNA by using Revert Aid Reverse Transcriptase and oligo-dT primer (Thermo Fisher Scientific, MA, USA). Primers for P2 receptors, EC, and SMC specific markers in the human and bovine system were designed and shown in the supplementary data available at http://dx.doi.org/10.1155/2014/413708. The RT-PCR conditions such as annealing temperature and magnesium concentration are given in the supplementary data as well. 1% of agarose gels were set up to evaluate the RT-PCR products. As positive control, RNA extracts from the cell lines HMEC-1, MG-63, C2C12, and U-87 MG were used for respective P2 receptor subtypes given in the supplementary data.

### 2.6. Western Blot Analysis

The proteins were extracted from the cells in a protein lysis buffer (Cell Signaling Technology, MA, USA) and subsequently centrifuged at 22,000 g for 5 min at 4°C to remove cellular debris. After boiling for 5 min, the lysate samples were separated by a 12% SDS-PAGE electrophoresis and electrotransferred to a PVDF membrane. The membrane was blocked in TBST containing 5% BSA and incubated with anti-P2X7, P2Y1, P2Y2, P2Y11, VEGFR2, VE-cadherin, PECAM-1, calponin, SMA-α, MYH-11 (1 : 500), or GAPDH antibodies (1 : 5,000) (Santa Cruz Biotechnology, CA, USA) overnight at 4°C. The membranes were washed three times with TBST and incubated with the secondary antibodies (1 : 5,000) (CALBIOCHEM, CA, USA) for 60 min at RT. After washing with TBST, immune-detection was accomplished by using the Luminata Forte Western HRP substrate (Merck Millipore, MA, USA) and images were taken using Bio-Rad Chemidoc system.

### 2.7. Immunofluorescence

The cells were fixed in 4% paraformaldehyde for 15 min. Cells were incubated with primary anti-VEGFR2, VE-cadherin, PECAM-1, calponin, SMA-α, MHY-11, P2X7, P2Y1, P2Y2, and P2Y11 (Santa Cruz Biotechnology, CA, USA) diluted in a ratio of 1 : 100 in antibody dilution buffer containing 1% BSA and 0.2% Triton-X-100 in PBS at 4°C overnight. After rinsing with PBS 3 times, cells were stained with FITC-labeled anti-goat or rabbit antibody, respectively (1 : 100) (Southern Biotech, AL, USA), at RT for 60 min. Cell nuclei were stained with DAPI (Sigma, MO, USA), and the cell cytoskeleton was labeled using rhodamine (1 : 2,000) (Life Technologies, CA, USA). After washing with PBS, fluorescent signals were analyzed with an Axio Observer D1 fluorescence microscope (Carl Zeiss, Germany) or a FW300 confocal fluorescent microscope (Olympus, Japan), respectively.

### 2.8. DiI-ac-LDL Uptake

ECs were incubated with 10 *μ*g/mL DiI-labeled acetylated-low density lipoprotein (DiI-ac-LDL) (Biomedical Technologies Inc. MA, USA) for 4 hours at 37°C and investigated with a fluorescent microscope (Carl Zeiss, Germany) at a wavelength of 565 nm. After staining with LDL, cells were fixed with 4% formaldehyde for 15 min and subsequently incubated with DAPI (1 : 10000 in PBS) and rinsed with PBS. Images were taken with an Axio Observer D1 fluorescent microscopy (Carl Zeiss, Germany).

### 2.9. Proliferation and Wound Assay

For the proliferation assay 20,000 ECs were seeded separately in each well of 12-well plates. ECs were grown in DMEM medium, in a SMC-conditioned medium in normal gravity (SMC CM + 1 g) and in a SMC-conditioned medium in simulated microgravity (SMC CM + MG) (see Section 2.4 for conditioned medium details). ECs incubated with the respective medium were subsequently obtained after 24 h and 48 h under normal gravity incubation and numbers were calculated. Similarly experiments were set for SMCs: SMCs were cultured in DMEM medium, in an EC-conditioned medium in normal gravity (EC CM + 1 g), and EC-conditioned medium in simulated microgravity (EC CM + MG) for 24 h and 48 h. Cell number in each well was counted.

For the wound assay ECs and SMCs (10,000/cm^2^) were seeded and grown to 80%–90% confluence. A straight scratch injury was made using a sterile 1 mL pipette tip on 6-well plates. The ECs were incubated for 24 h at 37°C in a CO_2_ incubator with normal DMEM medium, SMC-conditioned medium in normal gravity (SMC CM + 1 g), and SMC-conditioned medium in simulated microgravity (SMC CM + MG). On the other hand SMCs were cultured in DMEM medium, EC-conditioned medium in normal gravity (EC CM + 1 g), and EC-conditioned medium in simulated microgravity (EC CM+MG). Hydroxyurea (5 mM) was added to inhibit cell proliferation. Images were taken using a phase contrast microscope (Carl Zeiss, Germany). The numbers of migrated cells in three individual areas were calculated and quantified using Image J software (NIH).

### 2.10. Statistical Analysis

Statistical analysis was applied for the experiments using the Microsoft Office program Excel 2010 and SPSS 12.0. Data are shown as means ± standard deviation. Experiments were repeated at least three times for three donors, which are given as *n* = number of experiments. The probability (*P*) value was calculated using LSH test to assess differences between two groups. Levels of significance were labeled as follows: *P ≤ 0.05, **P ≤ 0.01, and ***P ≤ 0.001. Significance was given with the appropriate number of asterisks or in numbers.

## 3. Results

### 3.1. Characterization of Primary ECs and SMCs from Bovine Aorta

**Figure 2:**
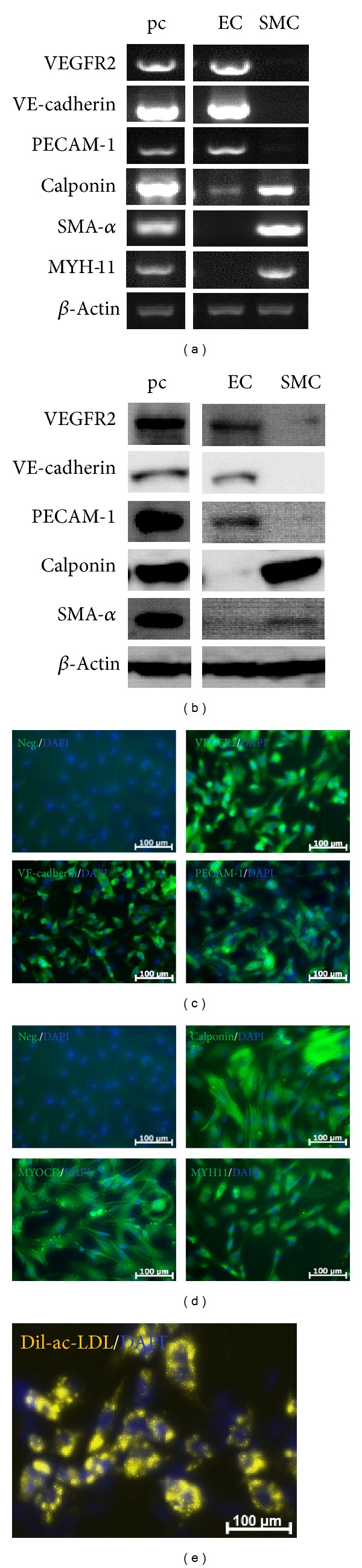
Characterization of Bovine Endothelial and Smooth Muscle Cells. The isolated cells were verified with the EC specific markers VEGFR2, VE-cadherin, PECAM-1 and the SMC specific markers calponin, SMA-α, MYH-11 by RT-PCR (a) and Western blot (b). GAPDH served as internal control. The endothelial (c) and smooth muscle cells (d) were also identified with the above-mentioned markers via fluorescent staining. The isolated endothelial cells were further examined for the typical endothelial activity of LDL up-take (e). All pictures are representative of one cow sample out of three.

The isolated ECs showed positive gene expression of endothelial cell markers VEGFR2, VE-cadherin, and PECAM-1, whereas SMCs positively expressed smooth muscle cell markers SMA-α, calponin, and MYH-11 (Figure 2(a)). Western blot experiments confirmed the gene expression data on the protein level. ECs positively expressed VEGFR2, VE-cadherin, and PECAM-1, while SMCs were positive for calponin, SMA-α, and MYH-11 (Figure 2(b)). Importantly both gene and protein data showed that ECs were negative for SMC markers except a weak band found in calponin. SMCs were negative for the three tested endothelial cell markers. These results indicate that isolated ECs and SMCs were without major cross contaminations. The fluorescent staining data further confirmed the results from the RT-PCR and Western blot analysis (Figures 2(c) and 2(d)). In addition, isolated ECs also showed the typical endothelial activity by uptaking LDL (Figure 2(e)).

### 3.2. P2 Receptor Expression in ECs after 24 h Simulated Microgravity with and without SMC-Conditioned Medium

**Figure 3:**
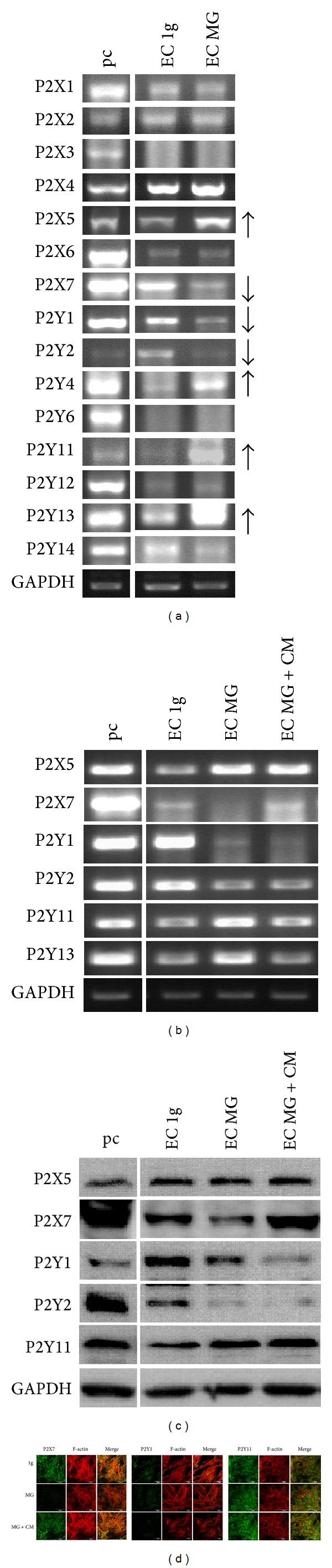
P2 Receptor Expression in Endothelial Cells after 24 h under Normal Gravity and Simulated Microgravity. All cells on the surface of flasks were isolated for RT-PCR. P2X5, P2Y4, P2Y11, P2Y13 were up-regulated and P2X7, P2Y1 and P2Y2 were down-regulated in the ECs after 24 h in the clinostat (a). Cells grown within 6 mm of the center had the optimal simulated microgravity condition and were therefore isolated to confirm the above P2 receptor alteration on the RNA (b) and protein (c) level after 24 h simulated microgravity with and without SMC-conditioned medium collected under normal gravity. P2X5 and P2Y11 were up-regulated in ECs but P2X5 up-regulation was not significant on protein level. P2X7, P2Y1, P2Y2 were down-regulated on both gene and protein level. The SMC-conditioned medium can compensate the decrease of P2X7 expression but cause no significant effect on the alteration of P2Y1, P2Y2 and P2Y11. The fluorescent staining confirmed the protein change of P2X7, P2Y1 and P2Y11 (d).

All fifteen P2 receptors were analyzed for their gene expression by RT-PCR. In the first experiment, RNA was collected in one set of clinorotation experiments from the whole culture flask. All P2 receptors were expressed in ECs with the exception of P2X3 and P2Y6. Next to this P2X5, P2Y4, P2Y11, and P2Y14 were upregulated, while P2X7, P2Y1, and P2Y4 were downregulated on the gene expression level in ECs under 24 h simulated microgravity condition (MG) induced by clinorotation if compared to ECs under normal gravity (1 g) (Figure 3(a)). In a further set of clinorotation experiments, the conditioned medium collected from SMCs grown under normal gravity condition (see Section 2.4) was added to ECs. For this experiment, only cells grown in the 6 mm diameter area of the center were taken to isolate RNA and protein. RT-PCR data showed that although the expression of P2X7 and P2Y1 was decreased after clinorotation P2X7 in ECs showed an increase on the gene level when cultured in SMC-conditioned medium. P2Y11 protein expression in ECs was upregulated and further increased also on the SMC conditioned medium compared to P2X7 (Figure 3(b)). Western blot and fluorescence confirmed the change of P2X7 on protein level (Figures 3(c) and 3(d)).

### 3.3. P2 Receptor Expression in SMCs after 24 h Simulated Microgravity with and without EC-Conditioned Medium

**Figure 4:**
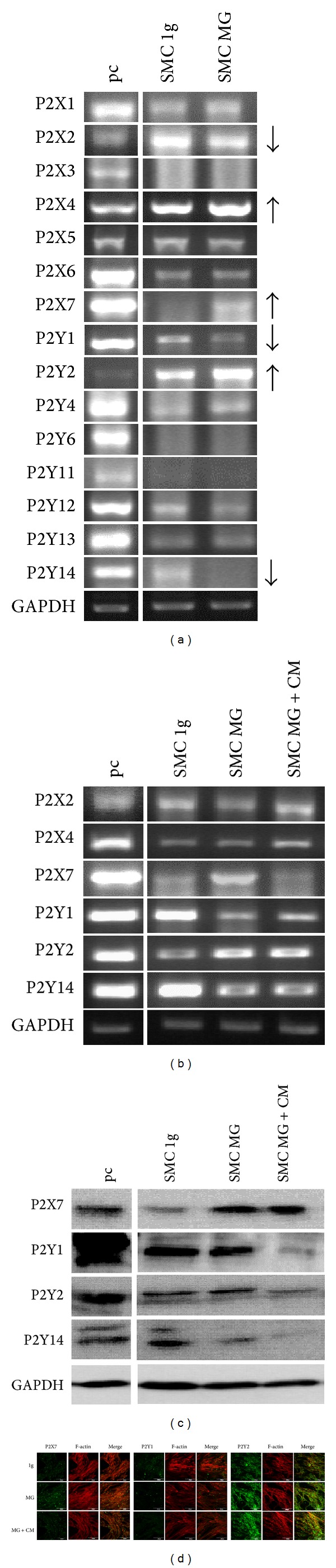
P2 Receptor Expression in Smooth Muscle Cells after 24 h under Normal Gravity and Simulated Microgravity. The experiments for SMC were performed similarly to those for the endothelial cells above. All cells on the surface of flasks were isolated for RT-PCR. P2X4, P2X7 and P2Y2 were up-regulated whereas P2X2, P2Y1 and P2Y14 were down-regulated in the SMCs after 24 h clinorotation (a). Cells grown within 6 mm of the center had the optimal simulated microgravity condition and were thus isolated to confirm the above P2 receptor alteration on both, RNA (b) and protein (c) level after 24 h clinorotation with and without EC-conditioned medium from normal gravity. P2X2 and P2X4 showed no significantly changed. P2X7 and P2Y2 was up-regulated, P2Y1 and P2Y14 were down-regulated. The EC-conditioned medium can compensate for the increase of P2X7 and P2Y2 expression, but no significant effect was observed on P2Y1 and P2Y14 (d).

Identical operational steps were undertaken to investigate SMCs under simulated microgravity. In SMCs, all P2 receptors were expressed except P2X3, P2X7, P2Y6, and P2Y11. After 24 h clinorotation, RT-PCR showed an increased gene expression of P2X4, P2X7, and P2Y2, whereas P2X2, P2Y1, and P2Y14 were downregulated in SMCs under simulated microgravity condition (MG) if compared to the SMCs under normal gravity (1 g) (Figure 4(a)). After adding EC-conditioned medium (see Section 2.4) within clinostat experiment, clinorotation induced an upregulation of P2X7 gene expression in SMCs as revealed by RT-PCR (Figure 4(b)). Interestingly, P2X7 showed a decreased gene expression after adding EC-conditioned medium compared to its increase without EC-conditioned medium under 24 h clinorotation. P2Y1 was upregulated in SMCs under simulated microgravity; however conditioned medium showed no effect on its expression. Gene level alterations of P2X7 and P2Y2 were confirmed on the protein level by Western blot or fluorescent staining; however, P2Y1 showed an increasing protein expression in simulated microgravity and with EC-conditioned medium (Figures 4(c) and 4(d)).

### 3.4. Proliferation and Migration of ECs Cultured with SMC-Conditioned Medium Collected under Normal Gravity and Simulated Microgravity

**Figure 5:**
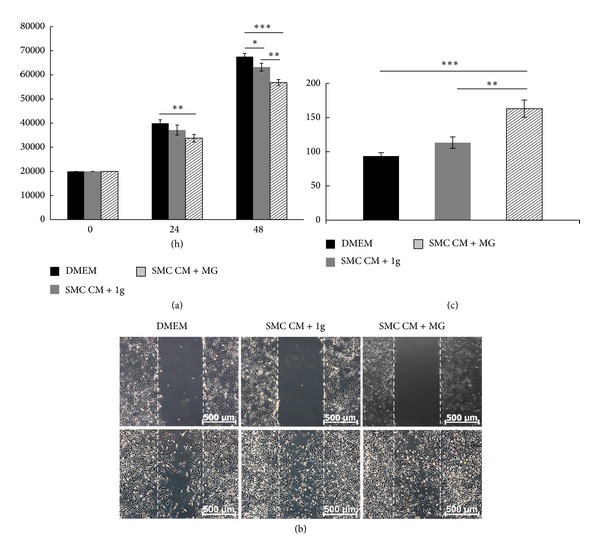
Effect of SMC-Conditioned Medium on EC Proliferation and Migration. To evaluate the EC proliferation, normal DMEM medium, and conditioned medium of SMCs after normal gravity (SMC CM + 1 g) and simulated microgravity (SMC CM + MG), was added for a 24 h and 48 h culture period. The conditioned medium from SMCs after normal gravity showed a decrease of EC numbers after 48 h. SMC-conditioned medium under simulated microgravity inhibited EC proliferation significantly after 24 h and 48 h incubation (a). To evaluate EC migration, ECs were scratched and normal DMEM medium, SMC-conditioned medium under normal gravity and under simulated microgravity was added for a 24 h culture period (b). Conditioned medium from SMCs under simulated microgravity (SMC CM + MG) enhanced EC migration significantly if compared to the normal DMEM medium and SMC-conditioned medium under normal gravity (c).

Conditioned medium from SMCs collected after 24 h normal gravity and after 24 h simulated microgravity was used to culture ECs evaluating the paracrine influence of SMCs on EC proliferation and migration. SMC-conditioned medium from normal gravity (SMC CM + 1 g) did not have a significant influence on EC proliferation after 24 h but caused a decrease of EC numbers after 48 h. SMC-conditioned medium collected after simulated microgravity (SMC CM + MG) inhibited EC proliferation significantly after both 24 h and 48 h, respectively (Figure 5(a)). To mimic a wound in the endothelium a straight scratch through the cells was set. The SMC-conditioned medium cultured under normal gravity (SMC CM + 1 g) and simulated microgravity (SMC CM + MG) condition was added to study EC migration capacity. The conditioned medium from MG enhanced EC migration after 24 h, and even more significantly after 48 h in the presence of hydroxyurea (Figures 5(b) and 5(c)). Figure 5 is a representative of example one of three cows. The numbers of proliferating and migrating ECs from the three individual cows are given in the supplementary data.

### 3.5. Proliferation and Migration of SMCs Grown in EC Derived Conditioned Medium Collected after 24 h under Normal Gravity and Simulated Microgravity

**Figure 6:**
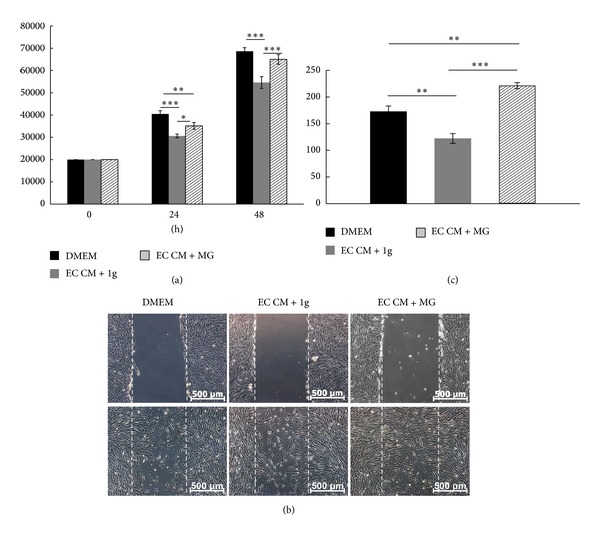
Effect of EC-Conditioned Medium on SMC Proliferation and Migration. To investigate the SMC proliferation, normal DMEM medium, and EC-conditioned medium under normal gravity (EC CM + 1 g) and simulated microgravity (EC CM + MG), was added and SMCs were subsequently incubated for 24 h and 48 h. Conditioned medium from EC grown under normal gravity (EC CM + 1 g) significantly inhibited SMC proliferation after 24 h and 48 h incubation and conditioned medium from EC grown under simulated microgravity (EC CM + MG) led to a significant decrease of SMC numbers after 24 h, which is not obvious after 48 h (a). To evaluate SMC migration, SMCs were scratched and cultured under normal DMEM medium, EC-conditioned medium under normal gravity and under simulated microgravity for 24 h (b). EC-conditioned medium under normal gravity (EC CM + 1 g) inhibited the SMC migration significantly. Whereas EC-conditioned medium under simulated microgravity (EC CM + MG) enhanced migrated SMC numbers (c).

Experiments with SMCs were performed in a comparable manner as described for ECs. The conditioned medium collected from EC grown under normal gravity (EC CM + 1 g) reduced the proliferation of SMCs. However, the conditioned medium collected from EC grown under simulated microgravity condition (EC CM + MG) compensated this effect (Figure 6(a)). Conditioned medium under simulated microgravity induced SMC migration after 48 h but inhibited it after 24 h (Figures 6(b) and 6(c)). Figure 6 is a representative of example one of three individual cows. The numbers of proliferating and migrating SMCs from the three individual cows are given in the supplementary data.

## 4. Discussion

**Figure 7:**
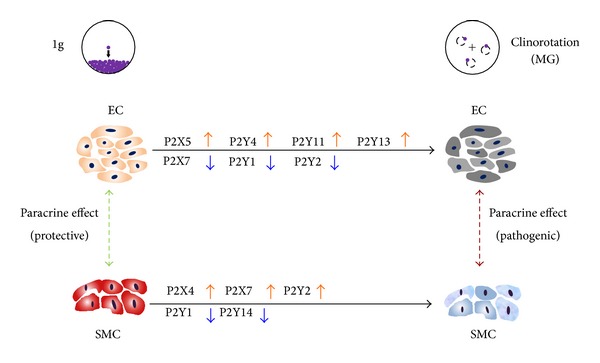
Scheme of P2 Receptor Alteration and the Postulated Paracrine Effect in ECs and SMCs under Simulated Microgravity. Several P2 receptor expressions were altered in ECs and SMCs under 24 h simulated microgravity condition using a clinostat. To point out that the expression P2X7 and P2Y2 was altered differentially between ECs and SMCs under simulated microgravity. Especially, the change of P2X7 in ECs was compensated under SMC-conditioned medium and vice versa. The conditioned medium collected under simulated microgravity showed the pathogenic influence of EC and SMC proliferation and migration if compared to condition medium from normal gravity.

In this study, we showed for the first time that several specific P2 receptor expressions were altered on gene and protein level after 24 h under simulated microgravity condition as shown in Figure 7. Culturing ECs with SMC-conditioned medium under normal gravity and vice versa can compensate the P2 receptor expression change such as P2X7.

Similar to the findings of Wang and colleagues [24], our data showed that ECs and SMCs expressed different P2 receptors on cell membrane. P2X4, P2Y1, P2Y2, and P2Y11 were predominantly expressed in ECs, while P2X1 and P2Y2 were strongly expressed in SMCs. Macrovascular and microvascular ECs have shown the several functional differences such as matrix metalloproteinase expression [25] and beta-adrenergic regulation of transendothelial permeability [26]. The expression of P2X3 and P2Y4 is low in bovine aortic ECs if compared to the control HMEC-1 which suggests that macrovascular ECs might differ in the P2 receptor expression pattern compared to microvascular ECs.

The P2 receptor expression patterns of ECs and SMCs have already been shown to play an important role in various cardiovascular functions. For example, in controlling vascular tone, ATP and UTP released from ECs act on P2Y1, P2Y2, and P2Y4 leading to the production of NO and subsequent vasodilatation. Simultaneously, ATP released by the sympathetic nerve acts on P2X1, P2X2, and P2X4, resulting in vasoconstriction [9]. We found that the expression of P2X2 and P2X4 in SMCs was significantly increased after clinorotation indicating to maybe more vasoconstriction. Next to this P2Y1 and P2Y2 expressions were decreased, which suggests NO production might decrease and cause less vasodilatation. Kang and colleagues found that 72 h exposure to clinorotation led to a decreased proliferation but increased the rate of apoptotic SMCs. Additionally, the SMC phenotype was induced and transferred from the contractive to the synthetic type [18]. Our data showed that the expression of P2X7 and P2Y2 was altered differentially between ECs and SMCs under simulated microgravity, which indicates that they could be the key P2 receptor subtypes responding to the change of gravity. To point out, P2X7 has an important role in cell apoptosis and can activate a series of downstream signals due to several protein kinase binding sites on its long intracellular tail [10]. A mechanical force such as shear stress can induce endothelial cell apoptosis that might be regulated through P2X7. It is of interest that P2X7, which was downregulated in ECs, was upregulated in SMCs. This change could be compensated by adding conditioned medium from the other cell type. Such compensation was not found in the other P2 receptor subtypes which might point to P2X7 as a major player in the interaction between ECs and SMCs in simulated microgravity with respect to apoptosis regulation.

Various proteins of endothelial cells are altered under real or simulated microgravity, such as F-actin [27], tubulin [28], cell adhesion molecules [16], integrins [27], eNOS [29], and iNOS [30]. In line with that ECs also showed a decreased proliferation rate, increased apoptosis [28, 31], and migration [29] in simulated microgravity. However, these data were observed based on cultured ECs as single cell type, either under real or simulated microgravity. An EC and SMC coculture model was successfully created via EC-conditioned medium culturing SMCs and vice versa. In addition to the paracrine effect on P2 receptor expression such as P2X7, our results showed that this effect found under simulated microgravity could influence EC or SMC behavior for cell proliferation and migration. SMC proliferation has been demonstrated to be a crucial process in atherosclerosis since migrated and proliferating SMCs form a major cell type in the plaque [32].

In healthy vessels ECs secrete cytokines that inhibit SMC proliferation and form a monolayer to block small molecules from blood that might cause SMC proliferation. We found that conditioned medium collected from ECs in normal gravity inhibited SMC proliferation, but conditioned medium collected from ECs after clinorotation was not able to do so. On the other hand, EC damage or dysfunction is one of the first steps during the development of pathological change in atherosclerosis. The conditioned medium of SMC grown in simulated microgravity reduced EC proliferation. Enhanced apoptosis was observed when only ECs were cultured in simulated microgravity by Infanger and coauthors [28]. They found several caspases such as caspase-3 and caspase-9 activated after simulated microgravity treatment. Apoptosis might be induced by activation of NF-*κ*B via the PI3K/Akt pathway [28, 31]. These data suggest that astronauts may be more prone to suffer from cardiovascular diseases such as atherosclerosis during space missions and paracrine effects between ECs and SMCs might be the key factors in this process. On the other hand migration of ECs is the first step in angiogenesis and a major factor in metastasis. In addition, it also plays an important role in restenosis in the vascular system after application of a stent. There are evidences that microgravity can promote angiogenesis in both macrovascular and microvascular ECs when only ECs were cultured under simulated microgravity [29, 30]. Our data showed an enhanced number of migrated ECs when cultured with SMC-conditioned medium derived after clinostat application, compared to the DMEM control and SMC-conditioned medium collected after 24 h exposure to normal gravity. This indicates the effect of microgravity might enhance the angiogenesis via both autocrine and paracrine signals.

Contradictory observations have been demonstrated in several publications; for example, EC migration increased in simulated microgravity both in this study and in the study of Siamwala and colleagues [30] while Versari and colleagues found a decreased EC migration under simulated microgravity [33]. One explanation for these findings might be that different endothelial cells were used such as primary endothelial cells from an artery or umbilical vein, or an endothelial cell line (EAhy926), which might give a different response due to its immortalization and thus prolonged time in culture. Another explanation could be that different devices were applied to simulate microgravity conditions such as the clinostat, the RPM, and the RWV. Different equipment might produce different qualities of microgravity as well as a different amount and quality of shear stress during rotation [20]. Clinorotation was shown to produce the lowest shear forces and the central area used in our study has an optimized simulated microgravity environment [23]. Furthermore, different ECs from different body parts were used. Macrovascular and microvascular ECs already revealed a difference in promoting angiogenesis under real/simulated microgravity conditions, which is regulated via the iNOS-cGMP-PKG pathway in macrovascular ECs but via eNOS-PI3K-Akt in microvascular ECs [29, 30]. Taken together, the simulated microgravity data independently of ground-based facility we use have to be approved and verified in real microgravity for a final statement on the outcome.

## 5. Conclusion

Our data show for the first time that P2 receptor gene and protein expression in both ECs and SMCs were altered under simulated microgravity. SMC-conditioned medium collected under simulated microgravity influenced some P2 receptor expressions as well as proliferation and migration of ECs and vice versa. Additionally, proliferation and migration of ECs and SMCs differed between conditioned medium collected under normal gravity and under simulated microgravity. These data suggest that the extracellular environment such as paracrine signals is an important factor and cannot be ignored considering the impact of microgravity on vascular cells. Since some P2 receptor artificial ligands are already applied as drugs for cardiovascular patients, specific P2 receptor ligands might be reasonable candidates to investigating their function for cardiovascular deconditioning under microgravity in the future.

## Supplementary Material

The table 1 is showed for the number of endothelial cell numbers culturing under DMEM, SMC CM+1g and SMC CM+MG conditions for 24h, 48h respectively. The figure 5(a) is presented as one example (Donor 1) of three individual cows.
The table 2 is showed for the number of migrated endothelial cell numbers culturing under DMEM, SMC CM+1g and SMC CM+MG conditions for 24h. The figure 5(b) is presented as one example (Donor 1) of three individual cows.
The table 3 is showed for the number of smooth muscle cell numbers culturing under DMEM, EC CM+1g and EC CM+MG conditions. The figure 6(a) is presented as one example (Donor 1) of three individual cows.
The table 4 is showed for the number of migrated smooth muscle cell numbers culturing under DMEM, EC CM+1g and EC CM+MG conditions. The figure 6(b) is presented as one example (Donor 1) of three individual cows.
The amplification conditions for each specific product were conducted as followed: the initial denaturation was performed at 94°C for 3 min and cyclic denaturation was run for 30 seconds. The cyclic annealing step was performed for 30 seconds with the respective annealing temperatures (see table 5). The target gene was elongated at 72°C for 45 seconds. The cyclic amplification was repeated with different numbers (see table 5). A final extension of 3 min at 72°C was set with subsequent cooling down to 4°C. GAPDH served as the housekeeping gene controls, which were adjusted to equal levels prior to the comparison of genes of interest. The RT-PCR product was evaluated with 1% agarose gel electrophoresis. The images of gels were taken with a Bio-Rad Chemidoc machine.
The cell line HMEC-1 and C2 were used as positive controls to confirm the EC and SMC specific markers, respectively. Since no single cell type can expresses all P2 receptor subtypes, different positive controls were used here: HMEC-1 for P2X3, P2X4, P2X5, P2X7, P2Y1, P2Y2, P2Y4, P2Y11, P2Y12; MG-63 for P2X6, P2Y6 and U-87 MG for P2X1, P2X2, P2Y13, P2Y14. The GAPDH of the positive control is only given for HMEC-1 as representative for all three positive controls.
