# Supplementary material for: The Influence of Simulated Microgravity on Purinergic Signaling Is Different between Individual Culture and Endothelial and Smooth Muscle Cell Coculture
Source: Biomed Res Int. 2014 Aug 28;2014:413708. doi: 10.1155/2014/413708 (PMC4163442; doi:10.1155/2014/413708)
Supplement: Supplementary file 1 — The table 1 is showed for the number of endothelial cell numbers culturing under DMEM, SMC CM+1g and SMC CM+MG conditions for 24h, 48h respectively. The figure 5(a) is presented as one example (Donor 1) of three individual cows. The table 2 is showed for the number of migrated endothelial cell numbers culturing under DMEM, SMC CM+1g and SMC CM+MG conditions for 24h. The figure 5(b) is presented as one example (Donor 1) of three individual cows. The table 3 is showed for the number of smooth muscle cell numbers culturing under DMEM, EC CM+1g and EC CM+MG conditions. The figure 6(a) is presented as one example (Donor 1) of three individual cows. The table 4 is showed for the number of migrated smooth muscle cell numbers culturing under DMEM, EC CM+1g and EC CM+MG conditions. The figure 6(b) is presented as one example (Donor 1) of three individual cows. The amplification conditions for each specific product were conducted as followed: the initial denaturation was performed at 94°C for 3 min and cyclic denaturation was run for 30 seconds. The cyclic annealing step was performed for 30 seconds with the respective annealing temperatures (see table 5). The target gene was elongated at 72°C for 45 seconds. The cyclic amplification was repeated with different numbers (see table 5). A final extension of 3 min at 72°C was set with subsequent cooling down to 4°C. GAPDH served as the housekeeping gene controls, which were adjusted to equal levels prior to the comparison of genes of interest. The RT-PCR product was evaluated with 1% agarose gel electrophoresis. The images of gels were taken with a Bio-Rad Chemidoc machine. The cell line HMEC-1 and C2 were used as positive controls to confirm the EC and SMC specific markers, respectively. Since no single cell type can expresses all P2 receptor subtypes, different positive controls were used here: HMEC-1 for P2X3, P2X4, P2X5, P2X7, P2Y1, P2Y2, P2Y4, P2Y11, P2Y12; MG-63 for P2X6, P2Y6 and U-87 MG for P2X1, P2X2, P2Y13, P2Y14. The G [file 413708.f1.pdf]

Supplemental data

Table 1. Number of Endothelial Cells after Incubation with SMC-Conditioned Medium after 24h and 48h Exposure in Normal Gravity and Simulated Microgravity.

	Medium	0h	24h	48h
EC (Cow 1)	DMEM	20000	39938 ± 1452	67500 ± 1369
	SMC CM+1g	20000	37125 ± 2054	63188 ± 1663
	SMC CM+MG	20000	33750 ± 1591	56813 ± 1281
EC (Cow 2)	DMEM	20000	40469 ± 1041	87500 ± 3536
	SMC CM+1g	20000	39047 ± 2585	82500 ± 2041
	SMC CM+MG	20000	32266 ± 1094	63125 ± 4270
EC (Cow 3)	DMEM	20000	37708 ± 1301	70208 ± 2818
	SMC CM+1g	20000	36875 ± 3903	63542 ± 955
	SMC CM+MG	20000	23958 ± 955	41458 ± 2009

Table 2. The Number of Migrated Endothelial Cells after Incubation with SMC-Conditioned Medium after 24h Exposure in Normal Gravity and Simulated Microgravity.

	Medium	24h
EC (Cow 1)	DMEM	94±5
	SMC CM+1g	113±8
	SMC CM+MG	163±13
EC (Cow 2)	DMEM	98±9
	SMC CM+1g	112±9
	SMC CM+MG	151±20
EC (Cow 3)	DMEM	86±5
	SMC CM+1g	109±4
	SMC CM+MG	138±17

Table 3. Number of Smooth Muscle Cells after Incubation with EC-Conditioned Medium after 24h Exposure in Normal Gravity and Simulated Microgravity.

	Medium	0h	24h	48h
**SMC (Cow 1)**	DMEM	20000	40500 ± 1472	68625 ± 1652
	EC CM+1g	20000	30625 ± 854	54625 ± 2689
	EC CM+MG	20000	35125 ± 1548	65000 ± 2160
**SMC (Cow 2)**	DMEM	20000	49594 ± 986	142625 ± 5441
	EC CM+1g	20000	40594 ± 1161	94500 ± 4041
	EC CM+MG	20000	47344 ± 1120	122500 ± 5152
**SMC (Cow 3)**	DMEM	20000	41500 ± 3000	102083 ± 5052
	EC CM+1g	20000	28167 ± 2566	54583 ± 1909
	EC CM+MG	20000	38167 ± 2021	87917 ± 2602

Table 4 The Number of Migrated Smooth Muscle Cells after Incubation with EC-Conditioned Medium after 24h Exposure in Normal Gravity and Simulated Microgravity.

	Medium	24h
**SMC (Cow 1)**	DMEM	173±10
	EC CM+1g	122±9
	EC CM+MG	221±6
**SMC (Cow 2)**	DMEM	169±7
	EC CM+1g	129±5
	EC CM+MG	208±15
**SMC (Cow 3)**	DMEM	147±11
	EC CM+1g	113±6
	EC CM+MG	188±15

Table 5 Primer List and RT-PCR Amplification Condition

Name	Forward Primer (5’→3’)<br>Reverse Primer (5’→3’)	Size [bp]	Ta [°C]	Mg^{2+} [mM]	Cycle
**P2X1**	TCT AYG AGA AGG GCT ACC AGA CC<br>TCC ACC TCC ACG GGG CAC CAG	361	60	2.5	45
**P2X2**	CCA TCA TCA CCA ARG TCA AGG<br>TTG GGG TAG TGG ATG CTG TT	391	53	2.5	45
**P2X3**	GCC GCT GCG TSA ACT ACA<br>GGT GGG RAT GAT GTT GAA CT	560	52	2.5	45
**P2X4**	TTC CTG TTC GAG TAC GAC ACG<br>GAA TAT GGG GCA GAA GGG ATC	659	56	2.5	40
**P2X5**	GTC ATC GCC AAG AAC AAG AAG GTG<br>CAG TCG GAA GAT GGG GCA GTA G	639	60	2	40
**P2X6**	GTT CTT CTT GGT GAC CAA CTT CC<br>CGG ATY CCA TAG AGC TTG AGC A	678	58	2	42
**P2X7**	GGT TGT AAA AAG GGA TGG WTG G<br>ACA GCA CTT GCA MCA GGG ATA A	723	55	3	45
**P2Y1**	CTT CCT GGG CAA CAG CGT GGC<br>CAG CAC CAA GGG GAC ACA GAA CAT	502	61	3	40
**P2Y2**	TGT GCC GCC TCA AGA CCT GGA A<br>GAA GGA GTA GTA GAG GGT GCG G	638	60	1.5	40
**P2Y4**	GAK TTC AAG TTC ATC CTG CTG<br>TGA GYC CAT AGC ARA CAA GAG T	574	52	2	45
**P2Y6**	CGG GCY GCC TGG CTA GTG TG<br>TTG GTG ATG TGR AAA GGC AGG AAG C	354	61	2	45
**P2Y11**	AGC GTC ATC TTC ATC ACC TG<br>CAT GTA GAG TAG MGG GTG GA	621	52	2	45
**P2Y12**	ATC GCT ACC AGA ARA CCA CCA GGC<br>CGG GCA AAA TGG AAW GGA ACA AAA CA	406	60	2.5	45
**P2Y13**	TGA GCA ACA AGG AAG CAA CAC CAT C<br>ACA CAA AGA AGA CAG CCA CGA CAA C	258	60	3	45
**P2Y14**	ATG TAC GTC AGC ATY GTG TTC TT<br>TAG GGG ATT CTG GCA ATR TGG T	455	56	2	45
**VEGFR2**	GAG AGG TGC TGC TYM GAT TT<br>GGA AGG AAC TCT CAT TAG GA	649	52	1.5	40
**PECAM-1**	GGT RAT AGC CCC RGT GGA TGA<br>TTG GCC TTG GCT TTC CTC AG	411	59	2.5	42
**VE-cadherin**	CTG CAT CCT CAC CAT CAC AGT<br>CTC GTA GCC GTA GAT GTG CAG	384	61	1	40
**Calponin**	RCA GAT GGG CAG CAA CAA GG<br>ATT TAT TGT GCT CCA GTG AAR TAG AA	443	51	3.5	35
**SMA-α**	CAG GGC TGT TTT CCC ATC<br>CCA TCT CCA GAG TCC AGC A	393	51	1.5	30
**MYH-11**	GGG CAG AGC AAA ATC TTC TT<br>GTT TCT TGA TCT TGG CCT CAG	658	51	2	42
**GAPDH**	CGT ATT GGG CGC CTG GTC ACC<br>GCC AGT GAG CTT CCC GTT CAG C	653	61	2.5	22

The amplification conditions for each specific product were conducted as followed: the initial denaturation was performed at 94°C for 3 min and cyclic denaturation was run for 30 seconds. The cyclic annealing step was performed for 30 seconds with the respective annealing temperatures (see table 5). The target gene was elongated at 72°C for 45 seconds. The cyclic amplification was repeated

with different numbers (see table 5). A final extension of 3 min at 72°C was set with subsequent cooling down to 4°C. GAPDH served as the housekeeping gene controls, which were adjusted to equal levels prior to the comparison of genes of interest. The RT-PCR product was evaluated with 1% agarose gel electrophoresis. The images of gels were taken with a Bio-Rad Chemidoc machine.

The cell line HMEC-1 and C2 were used as positive controls to confirm the EC and SMC specific markers, respectively. Since no single cell type can expresses all P2 receptor subtypes, different positive controls were used here: HMEC-1 for P2X3, P2X4, P2X5, P2X7, P2Y1, P2Y2, P2Y4, P2Y11, P2Y12; MG-63 for P2X6, P2Y6 and U-87 MG for P2X1, P2X2, P2Y13, P2Y14. The GAPDH of the positive control is only given for HMEC-1 as representative for all three positive controls.
